# Myxofibrosarcoma involving brachial plexus diagnoses by contrast-enhanced ultrasound: A case report

**DOI:** 10.1097/MD.0000000000036626

**Published:** 2023-12-15

**Authors:** Weijie Liu, Yumei Yan, Xiaohang Wu, Xiukun Hou, Xiaomeng Qu

**Affiliations:** a Department of Ultrasound, First Affiliated Hospital of Dalian Medical University, Dalian, Liaoning, China.

**Keywords:** brachial plexus, contrast-enhanced ultrasound, myxofibrosarcoma, perfusion pattern

## Abstract

**Rationale:**

Myxofibrosarcoma most commonly arises as a slowly enlarging, painless mass. We describe an unusual case of low-grade myxofibrosarcoma in the axillary fossa, which infiltrated the brachial plexus, axillary artery, and axillary vein, causing severe pain. The low incidence and complex anatomical structure make imaging examination and surgery face great challenges. To the best of our knowledge, such presentation of a low-grade myxofibrosarcoma that showed an extreme infiltrative growth pattern and presented severe pain has not been reported before.

**Patient concerns:**

We reported a case of low-grade myxofibrosarcoma developed around the axillary neurovascular bundle, with multiple peripheral metastases in an 87-year-old male. Physical examination revealed a mass on the right axillary fossa measuring 5 × 4 cm. The patient underwent computed tomography but no definite diagnosis was obtained. Because he had claustrophobia and could not perform MRI examination. Thus, he underwent conventional ultrasound and contrast-enhanced ultrasound. Ultrasonic examination not only accurately determines the invasion scope of the tumor, but also clearly shows that the nerve has suffered from the invasion of the exogenous tumor and multiple metastatic foci around it. The contrast enhancement mode of the tumor showed centripetal high-enhancement, uneven internal enhancement, visible enhanced bridge, and non-enhancing central area.

**Diagnoses:**

Combined with the results of conventional ultrasound and contrast-enhanced ultrasound, we highly suspected it to be soft tissue sarcoma, giving strong clinical assistance.

**Interventions:**

Given the risk of sarcoma implantation along the needle track and the underestimation of tumor malignancy, an excisional biopsy was considered the most practical choice to avoid unnecessary pain and potential implantation.

**Outcomes:**

The patient underwent surgery and a histopathological examination of the lesion confirmed it as low-grade myxofibrosarcoma.

**Lessons subsections:**

This report describes a rare case of myxofibrosarcoma of the axillary fossa. High-resolution ultrasound is increasingly used for the initial assessment of soft-tissue masses. However, there are few reports about the ultrasound and contrast-enhanced ultrasound examinations of myxofibrosarcoma. Accurate preoperative diagnosis and proper treatment strategies are critical in managing patients with myxofibrosarcoma. Our case may provide diagnosis experiences and will help better understand and treat this disease.

## 1. Introduction

Myxofibrosarcoma (MFS) is a fibroblast-derived sarcoma comprising approximately 5% to 10% of all malignant soft tissue tumors.^[1]^ MFS can occur at various ages but predominantly affects the extremities of elderly individuals aged 60 to 80.^[2]^ Superficial fascia of the trunk or extremities is the primary site for 80% to 90% of MFS cases, while only 8% to 10% occur in deeper locations.^[3]^ While magnetic resonance imaging (MRI) is considered the imaging modality of choice for myxofibrosarcoma,^[4]^ ultrasound imaging is often used due to the superficial location of the tumor. However, there are limited reports on the ultrasound (US) and contrast-enhanced ultrasound (CEUS) examinations of myxofibrosarcoma. Herein, we present a case of low-grade MFS involving the axillary neurovascular bundle, with multiple peripheral metastases, and describe its ultrasound features and perfusion pattern.

## 2. Case presentation

**Figure 1.**
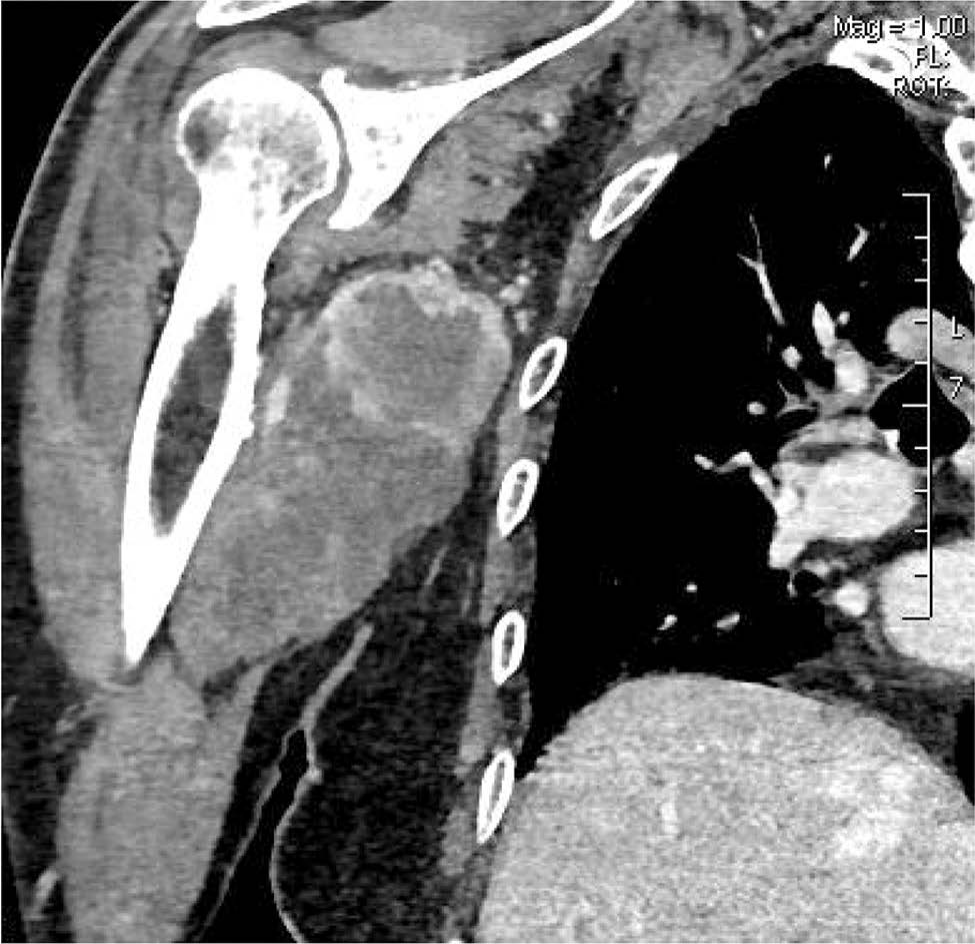
Contrast-enhanced computed tomography of the right axillary: a mixed density mass with rim enhancement.

An 87-year-old male patient presented with the discovery of an egg-sized mass in the right axillary fossa. Two months later, the patient began experiencing severe pain and numbness in the upper arm, which radiated to the forearm and persisted even at rest. The patient had a past medical history of diabetes and hypertension, and laboratory tests revealed normal results, except for an increased C-reactive protein level (15.8 mg/L). Physical examination revealed a clear and hard mass measuring 5 × 4 cm in the right axillary fossa, with poor mobility and no obvious adhesion to the skin. Computed tomography showed a lesion with rim enhancement, clear borders, and a maximum cross-sectional area of approximately 6.8 × 6.3 cm. The right brachial artery and its small branches were visible at the inner edge of the mass, and the local blood vessels became thinner (Fig. 1). Due to claustrophobia, an MRI examination was not feasible for the patient. Therefore, conventional ultrasound and CEUS were performed using ultra-high frequency (15 MHz), high-frequency (9 MHz), and low-frequency (4 MHz) linear transducers to accommodate the large size and depth of the mass. Subsequently, CEUS was utilized to assess the perfusion pattern of the tumor.

**Figure 2.**
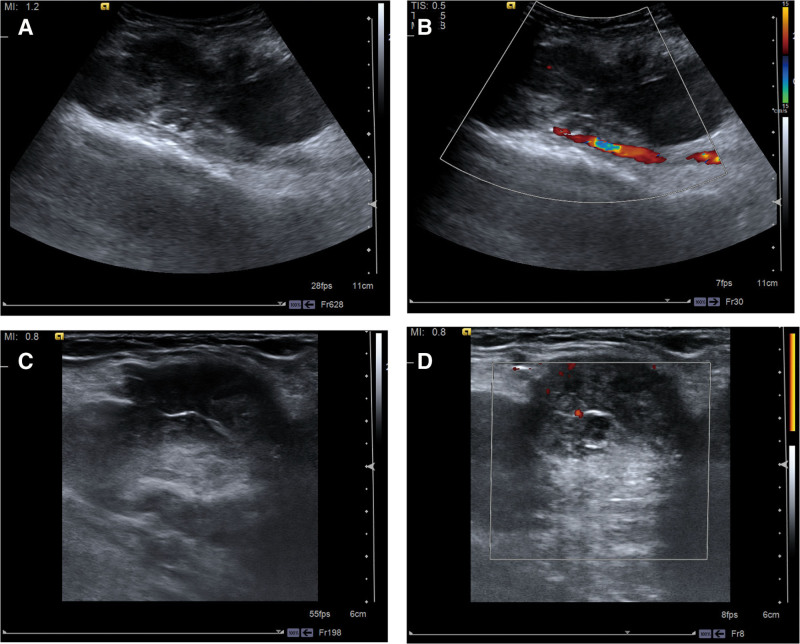
Ultrasound showed a heterogenous mass in the right axillary: (A) Gray-scale sonography of the right axillary evaluated by convex probe; (B) Color Doppler showing an arterial vessel nearby the mass; (C) Gray-scale sonography of the right axillary evaluated by linear probe; (D) Power Doppler shows the presence of vessels within the mass.

**Figure 3.**
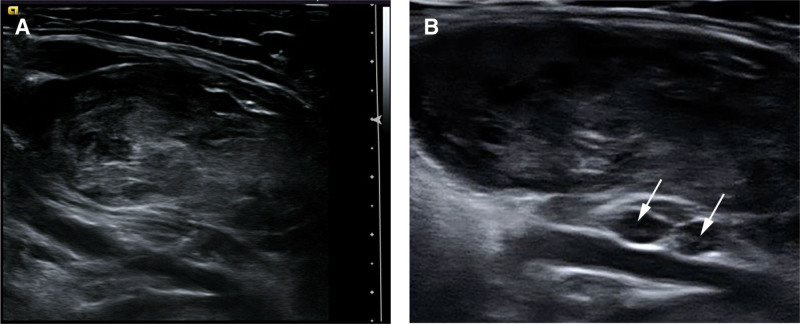
The tumor was large, and the nerves had been invaded: (A) The mass developed around the axillary neurovascular bundle; (B) The median nerve has been invaded (white arrows).

Conventional ultrasound revealed a heterogeneous mass measuring 123.3 × 66.2 × 50.2 mm in size adjacent to the humerus. The mass exhibited an irregular shape, clear boundaries, and uneven internal echo, with minimal blood flow signals in and around the tumor (Fig. 2). Numerous smaller homogeneous hypoechoic masses with indistinct boundaries were observed in the tumor’s vicinity involving the brachial artery and axillary artery adventitia, with the size 4 × 3 mm. It was considered metastatic lymph nodes based on ultrasound findings. The axillary artery and the unclear demarcation between the adventitia of the axillary artery and the mass were observed within the deep tumor. The axillary vein displayed interruption at the level of the deep tumor, with an unclear boundary and absent blood flow. Additionally, a hypoechoic mass surrounding the median nerve with detectable dotted blood flow (Fig. 3) and an elliptical hypoechoic mass adjacent to the ulnar nerve (measuring 8.9 × 8.7 × 6.9 mm) were visualized. The tumor severely impeded the radial nerve and medial cutaneous nerve of the forearm, making them unrecognizable by ultrasound.

**Figure 4.**
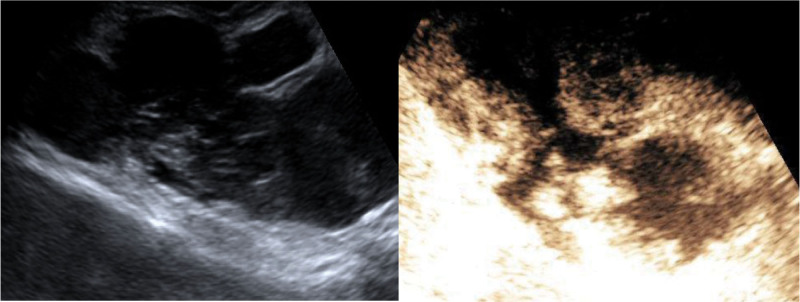
CEUS of the mass: 17 seconds after the injection of contrast agent evaluated by the convex probe. CEUS showed a high-enhancement centripetal area, uneven internal enhancement, visible enhanced bridge, and non-enhanced central area. CEUS = contrast-enhanced ultrasound.

**Figure 5.**
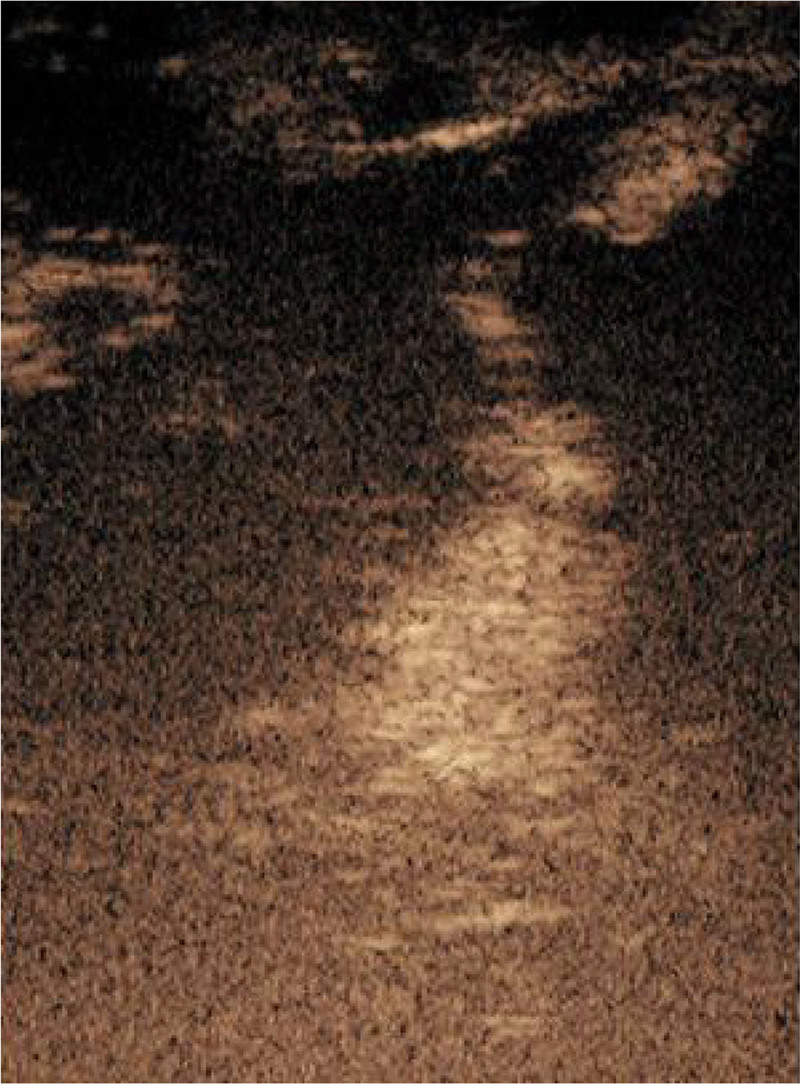
The 30 seconds after the linear probe was used to evaluate the injection of the contrast agent.

In CEUS, an MI setting of 0.08 was applied. A total of 4.8 mL of contrast agent (SonoVue®; Bracco, Dalian, Liaoning, China) was administered intravenously in 2 bolus doses of 2.4 mL, with the second dose given 15 minutes after the first. A saline flush of 5ml followed each dose, and the perfusion of the tumor was recorded in a continuous 3-minute movie loop. The contrast enhancement mode of the tumor demonstrated centripetal high-enhancement, uneven internal enhancement, a visible enhanced bridge, and a non-enhancing central area (Figs. 4 and 5). The enhancing tumor displayed wash-in after 13 seconds and wash-out after 3 minutes and 26 seconds.

**Figure 6.**
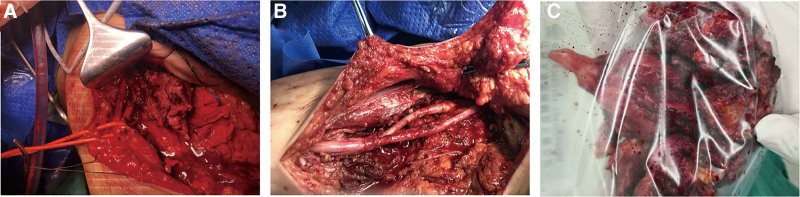
The photograph was taken during the operation: (A, B) the tumor was an infiltrative growth of neurovascular bundles with multiple scattered metastatic foci around it; (C) Excised tissues.

The initial suspicion of soft tissue sarcoma is high. Given the risk of sarcoma implantation along the needle track and the underestimation of tumor malignancy, an excisional biopsy was considered the most practical choice to avoid unnecessary pain and potential implantation.^[5]^ During surgery, the tumor was found surrounding the neurovascular bundle in the axillary region. Its distal end extended into the upper-third of the upper arm, penetrating deep into the proximal axilla. The superior border abutted the clavicle, the posterior aspect was located within the latissimus dorsi tendon, and the inner edge was in the chest wall. The tumor exhibited infiltrative growth along the neurovascular bundles, with multiple scattered metastatic foci (Fig. 6). Given the patient’s preference for pain resolution as the primary goal, palliative surgery was performed, removing the posterior bundle, the medial bundle, and a portion of the lateral bundle of the brachial plexus. The preserved nerves and surrounding muscle tissue were inactivated and rinsed with absolute ethanol. While a gross margin was achieved during surgery, no pathological examination of the surgical margin was performed.

**Figure 7.**
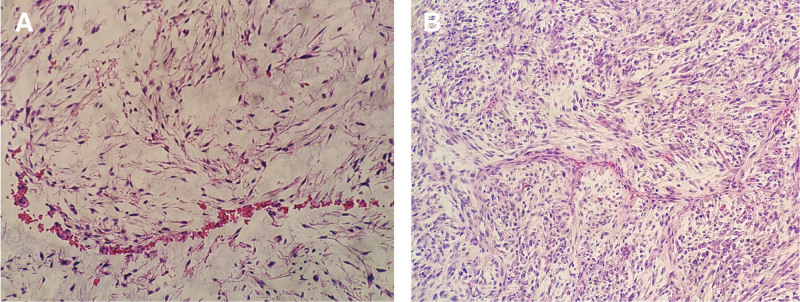
Hematoxylin and eosin stain: (A, B) High-power magnification view showing typical features of myxofibrosarcoma. The cellular proliferation of spindle cells containing large, elongated, hyperchromatic, and irregularly shaped nuclei is shown in a fibrous and myxoid stroma. Curvilinear vessels in the myxoid stroma with perivascular condensation of the tumor cells (× 200).

Histopathological examination revealed a tumor composed of spindle cells with localized interstitial myxoid degeneration accompanied by curvilinear small capillaries. Some areas showed a high density of tumor cells with hyperchromatic and irregularly shaped nuclei. On the contrary, other areas exhibited abundant myxoid matrix with necrotic regions present (Fig. 7). The immunohistochemical results demonstrated focal positivity for Desmin, negative staining for NF and S-100, positive staining for SMA and CD56, negative staining for CD34 and MUC4, and a high Ki-67 proliferation index of 50%. Based on histopathological and immunohistochemical findings, a diagnosis of low-grade MFS was established. The patient declined postoperative radiation therapy due to advanced age and poor general health. A computed tomography scan showed a local recurrence in the right axilla two months after surgery. Unfortunately, the patient passed away four months after the surgical intervention.

## 3. Discussion and conclusions

In 1996, Mentzel et al^[6]^ classified myxofibrosarcoma into low, intermediate, and high-grade categories. The local recurrence rate of MFS ranges from 16% to 57%.^[7]^ The propensity of MFS to spread along vascular and fascial planes contributes to the high rates of local tumor recurrence. The standard treatment for MFS typically involves wide local excision followed by selective postoperative radiotherapy or chemotherapy. Accurate preoperative diagnosis and appropriate treatment strategies are crucial for managing patients with MFS. Therefore, a complete imaging analysis of each case is essential to improve our understanding and management of this disease.

Imaging examinations play a crucial role in the diagnosis of MFS. Currently, MRI is commonly utilized in clinical practice. The “tail sign” observed on MRI is considered MFS’s most characteristic imaging feature, seen in 64% to 81% of MFS cases, particularly those located superficially.^[8]^ However, the tail sign can also present in patients with other sarcomas and benign soft tissue tumors.^[9]^ This tumor margin alteration can also be visualized by ultrasound, demonstrating a plate-like extension infiltrating the surrounding tissue.^[10]^ Ultrasound is increasingly used to assess soft tissue masses for initial assessment due to its convenience, noninvasiveness, and absence of radiation.^[11]^ Conventional ultrasound, when combined with CEUS, can provide additional diagnostic value by evaluating the perfusion pattern of soft tissue tumors.

Loizides identified four perfusion patterns of CEUS in soft tissue tumors^[12]^: P1 (non-enhancing mass or only rim-enhancement of the surrounding pseudo-capsule), P2 (peripherally enhancing mass with a non-enhancing central area), P3 (diffusely enhancing mass with scattered non-enhancing areas and/or enhancement bridges), and P4 (completely homogeneously enhancing mass). CEUS shows that 80% of P3 may be malignant, and the sensitivity and specificity of P3 mode are 89% and 78%, respectively. In our case, the CEUS mode of MFS is also shown as a P3 perfusion pattern. Demarchi suggests that the CEUS pattern is the most useful factor in diagnosing soft tissue tumors, with similar specificity and sensitivity to MRI.^[13]^

In this particular case, the ultrasound revealed a heterogeneous mass, with the hyperechoic part representing the tumor parenchyma and the hypoechoic part composed of mucus with high water content and necrotic areas. The low-grade MFS predominantly exhibited mucus with a low cell density. The sparsely distributed tumor cells within the mucus background were primarily along curvilinear blood vessels, with necrosis present. These findings correspond to the markedly enhanced bridges and non-enhancing areas observed on CEUS. Considering the pathophysiological characteristics of soft tissue tumors, evaluating soft tissue tumors using the perfusion pattern of CEUS holds significance.

MRI is used for diagnosing tumors by observing their signal and enhancement level, while CEUS is used to observe the blood perfusion pattern of soft tissue masses. CEUS has a high imaging frame rate, enabling real-time dynamic observation of microvascular perfusion, thereby improving the detection rate of lesions and the specificity and sensitivity of ultrasound examinations.^[14]^ Furthermore, its contrast perfusion pattern can further provide hints regarding the nature (benign or malignant) of the lesion. Compared to MRI, ultrasound and CEUS offer flexible positioning and can scan lesions in any section. CEUS surpasses 2D ultrasound in displaying blood perfusion and identifying necrotic areas more accurately. Furthermore, CEUS can facilitate the precise identification of necrotic regions, guiding representative sampling during fine needle aspiration biopsy of soft tissue lesions.^[15]^

In conclusion, CEUS is pivotal in elucidating tumor perfusion patterns, aiding preoperative differential diagnosis, guiding surgical approaches and treatment plans, and facilitating postoperative follow-up and recurrence studies.

## Acknowledgments

We would like to acknowledge the reviewers for their helpful comments on this paper.

## Author contributions

**Writing – original draft:** Weijie Liu，Xiaohang Wu

**Writing – review & editing:** Weijie Liu，Xiaohang Wu, Yumei

Yan, Xiukun Hou, Xiaomeng Qu.
